# Editorial: Benchmarks and good practices in bioinformatics

**DOI:** 10.3389/fbinf.2026.2005704

**Published:** 2026-10-07

**Authors:** Bruno César Feltes, Renato Augusto Corrêa dos Santos, Hugo Verli

**Affiliations:** 1 Department of Biophysics, Institute of Biosciences, Federal University of Rio Grande do Sul, Porto Alegre, Brazil; 2 Department of General and Applied Biology, Institute of Biosciences, São Paulo State University, Rio Claro, Brazil; 3 Center of Biotechnology, Federal University of Rio Grande do Sul, Porto Alegre, Brazil; 4 Brazilian Biosciences National Laboratory, Brazilian Center for Research in Energy and Materials (CNPEM), Campinas, Brazil

**Keywords:** benchmarks, bioinformatics, database, machine learning, omics, systems biology

## 1 Introduction

Bioinformatics has become an essential component of modern biological research, providing the computational support required to transform increasingly large and complex biological data into meaningful biological information. However, the rapid expansion of analytical tools, workflows, and new software has created a convoluted scenario, where both newcomers and veterans can become overwhelmed. In this sense, newcomers will struggle to find the best approach and navigate the massive sea of tools, pipelines, opinions, and databases available. On the other side, veterans often struggle to keep up with new tools and databases, eventually relying on their most well-known tools to run analyses.

Both issues are significant challenges in the field because different methodological choices can produce substantially different results from the same or comparable biological data. Consequently, increasingly sophisticated computational methods do not necessarily translate into more reliable biological information. Benchmarking has therefore become a fundamental component of bioinformatics, providing a useful comparison for determining how analytical choices, dataset characteristics, parameterization, and computational implementations influence the results researchers ultimately interpret. Benchmarking is a well-known way to compare analytical choices, tools, and methods, and it is used across many fields in molecular biology and bioinformatics (Guala and Sonnhammer, 2017; Warwick Vesztrocy and Dessimoz, 2020; Li et al., 2021; Luecken et al., 2022; Sarthak et al., 2023; Chen et al., 2025; Gong et al., 2025).

Importantly, meaningful benchmarking cannot be reduced to the idea of just identifying the tool that achieves the highest value for a particular performance metric. Computational methods operate within biological and analytical contexts, and their behavior may change with dataset composition, input size, sequencing characteristics, representation strategies, or even how the underlying biological question is defined. A method that performs well under one benchmark may therefore not be the most appropriate choice under different experimental conditions. Hence, meaningful benchmarking goes beyond metrics and must consider the biological question at hand.

Moreover, technically acceptable outputs may differ considerably in their biological informativeness and interpretability. Benchmarking should thus be understood not as a competition among tools, but as a means of defining their behavior, limitations, and appropriate domains of application for each research scenario. In this sense, dataset construction, software version and environments, workflow architecture, availability of meaningful documentation, parameter reporting, validation procedures, and usability all influence whether an analysis can be reproduced and reused.

As bioinformatics becomes accessible to a broader community, these principles become particularly important, since methodological complexity and inadequate software design can create substantial barriers for researchers who are not computational specialists.

This Research Topic includes seven articles that debate many aspects of this complex, fast-paced, and ever-adaptable field.

## 2 The research topic

García-Vinuesa et al. present a protein-specific workflow for reproducible machine-learning benchmarking, emphasizing data curation, versioned artifacts, interfaces, reproducible execution, and transparent evaluation. The authors highlight how protein-specific factors, such as evolutionary relationships, class construction, structural predictions, and potential pretraining contamination, can substantially influence benchmark interpretation, and illustrate the workflow through an antimicrobial peptide classification case study.

Oliveira et al. benchmark 12 widely used GO overrepresentation tools using controlled and experimental datasets of different sizes. Despite relying on the same enrichment strategy and knowledge base, the tools produced markedly heterogeneous biological outputs, differing in term specificity, target-pathway prioritization, stringency, and sensitivity to input size. The study demonstrates that conventional performance metrics alone may inadequately capture the biological informativeness and interpretability of functional enrichment results.

Maritan and Stewart provide an introduction to nucleic acid-based omics designed for early-stage researchers. The review organizes genomics and transcriptomics around their data products, analytical goals, and recurring modular workflow steps. The article provides a theoretical foundation intended to make increasingly complex omics workflows more accessible and interpretable to new users.

Leoni et al. showed the importance of context-specific benchmarking using SARS-CoV-2 subgenomic RNA detection as a case study. Across synthetic and real-world datasets, performance varied substantially according to sequencing strategy, mutation profile, read length, aligner, and primer design. The results show that bioinformatics workflow selection cannot be dissociated from experimental context and support benchmarking as a prerequisite for reliable and reproducible genomic analyses.

Pérez-Rodríguez et al. discuss bioinformatics reproducibility from a software-engineering perspective, adapting SOLID principles to workflow management systems and pipeline implementation. They debate explicit task interfaces, separation of computational and adaptation components, systematic validation, comprehensive testing, and continuous integration. The authors link these practices to FAIR-oriented software guidelines and provide a practical workflow to improve pipeline robustness and long-term reproducibility.

Fragkouli et al. introduce Synth4bench, a pipeline for generating controlled synthetic datasets to benchmark tumor-only somatic variant callers. Evaluation of five widely used callers revealed substantial performance differences associated with sequencing parameters and algorithmic design, with indel and low-allele-frequency detection remaining particularly challenging. The study demonstrates that no single caller performs optimally across all conditions, highlighting the importance of matching sequencing configurations and variant-calling algorithms to analytical objectives.

Finally, Patel et al. examine usability barriers novice genomic researchers encounter through literature analysis and semi-structured interviews. Major obstacles included inadequate documentation, limited accessible guidance, installation and dependency difficulties, and inconsistent error or progress reporting. The authors developed an evaluation rubric for bioinformatics software, showing user-centered design as an important but frequently overlooked component of tool development.
